# Imaging of electric and magnetic fields near plasmonic nanowires

**DOI:** 10.1038/srep22665

**Published:** 2016-03-07

**Authors:** I. V. Kabakova, A. de Hoogh, R. E. C. van der Wel, M. Wulf, B. le Feber, L. Kuipers

**Affiliations:** 1 FOM Institute AMOLF, Science park 104, 1098 XG Amsterdam, The Netherlands; 2 Blackett Laboratory, Imperial College London, London SW7 2AZ, United Kingdom; 3 Institute of Science and Technology Austria, Am Campus 1, A-3400 Klosterneuburg, Austria; 4 Optical Materials Laboratory, ETH Zurich, 8092 Zurich, Switzerland

## Abstract

Near-field imaging is a powerful tool to investigate the complex structure of light at the nanoscale. Recent advances in near-field imaging have indicated the possibility for the complete reconstruction of both electric and magnetic components of the evanescent field. Here we study the electro-magnetic field structure of surface plasmon polariton waves propagating along subwavelength gold nanowires by performing phase- and polarization-resolved near-field microscopy in collection mode. By applying the optical reciprocity theorem, we describe the signal collected by the probe as an overlap integral of the nanowire’s evanescent field and the probe’s response function. As a result, we find that the probe’s sensitivity to the magnetic field is approximately equal to its sensitivity to the electric field. Through rigorous modeling of the nanowire mode as well as the aperture probe response function, we obtain a good agreement between experimentally measured signals and a numerical model. Our findings provide a better understanding of aperture-based near-field imaging of the nanoscopic plasmonic and photonic structures and are helpful for the interpretation of future near-field experiments.

In the past few decades the study of the light field near nanophotonic structures has evolved into an independent research area, revealing new fundamental aspects of light-matter interaction at the nanoscale and propelling research in various applied fields of classical and quantum optics^1^,^2^,^3^,^4^,^5^,^6^. Near fields are difficult to image as they are evanescent in nature and decay within 10 s to 100 s of nanometers away from the surface, so that they cannot be directly viewed by a photodiode or a camera placed above the sample. In one of the near-field measurement techniques, known as near-field scanning optical microscopy (NSOM)^7^, the detection is usually done by frustrating the evanescent field and converting it into far-field radiation by means of a scattering probe (s-NSOM) or a light-collecting aperture probe (a-NSOM). In any case, a probe is brought close to the surface of the structure and scanned across to map the near-field distribution. Typically such probes have dimensions of a fraction of a wavelength, enabling super-resolution imaging.

Near-field imaging is a particularly important characterization method for objects of a certain type: those with a nanoscopic size, and those that are intrinsically lossy^8^,^9^. This is because many other measurement types, e.g. transmission measurement, are not applicable in this situation. It is, however, well known that the interpretation of an NSOM measurement is not always a straightforward task and several common artifacts of the near-field imaging have been previously pointed out^10^. It is therefore crucial to understand the process of the image formation for a variety of nanophotonic structures of different complexity and sizes.

Plasmonic nanostructures, being deeply-subwavelength and lossy, are commonly investigated in the near-field regime^11^,^12^,^13^. So far several geometries, including plasmonic wires and tapers^13^,^14^,^15^, nanoparticles^16^ and holes^17^,^18^,^19^,^20^, have been studied using NSOM. In these studies the signal collected by the near-field aperture probe was always attributed to the electric field. However, it has recently become apparent that the aperture probe of the near-field microscope is sensitive not only to the electric field but also to the magnetic field^21^,^22^,^23^,^24^,^25^,^26^,^27^. A study performed on photonic crystal waveguides (PhCWs) has shown that the probe sensitivity to the electric field component near a PhCW is approximately the same as that to the magnetic field. In addition, the electric and magnetic field patterns in this study are found to evolve differently with distance^21^. It is therefore important to treat information obtained through the near-field imaging with care and recognize the potential contributions of both fields in the measured signals.

In this article, we present a detailed study of the image formation in the a-NSOM measurement of plasmonic nanowires. Our study exploits the optical reciprocity theorem, the approach which we have previously used for PhCWs to successfully predict the detection of electric and magnetic components of the near field^21^. By considering the electric and magnetic field components of the nanowire mode as well as the complex response function of the aperture probe, we are able to reproduce the measured data with a good agreement. As for PhCWs, here we find that the probe sensitivity to the electric field is approximately equal to its sensitivity to the magnetic field. This leads us to the conclusion that the signal measured in the near-field microscopy experiments on plasmonic nanostructures is a combination of both fields, electric and magnetic, in other words the magnetic field cannot be ignored.

Next we analyze how the probe’s geometry influences the outcome of the imaging process. Two features need to be considered: (1) the complex nature of the probe’s response, i.e. the probe cannot be approximated as a simple filter, and (2) the geometrical size of the probe’s aperture being a factor of 2 larger than the nanowire width. As a result, the signal collected by the probe does not represent the electro-magnetic field of the nanowire mode at a single location, e.g. at the center of the aperture, but is proportional to the overlap integral of the local field and the probe’s response function. This makes the separation of the measured signals into individual electric and magnetic components non-trivial and suggests the need for a higher resolution near-field imaging technique. Our findings present a deeper understanding of the image formation in the aperture-type NSOM at the resolution limit and are valuable for future developments in the near-field imaging of photonic and plasmonic objects of nanoscopic sizes.

## Results

### Near-field microscopy of plasmonic nanowires

In this work we use a state-of-the-art scanning near-field microscope, operating in collection mode. This setup is capable of measuring the complex electro-magnetic field in planes above the sample by raster-scanning the aperture probe and recording the signal collected by the probe^26^. The probe consists of an optical fiber taper with a diameter of approximately 200 nm and a 150 nm thick aluminum coating. Such a probe diameter of *λ*/7 typically presents a trade-off between the resolution and the signal-to-noise ratio of the measurement. The resolution of this imaging technique is mostly determined by the probe’s aperture.

**Figure 1:**
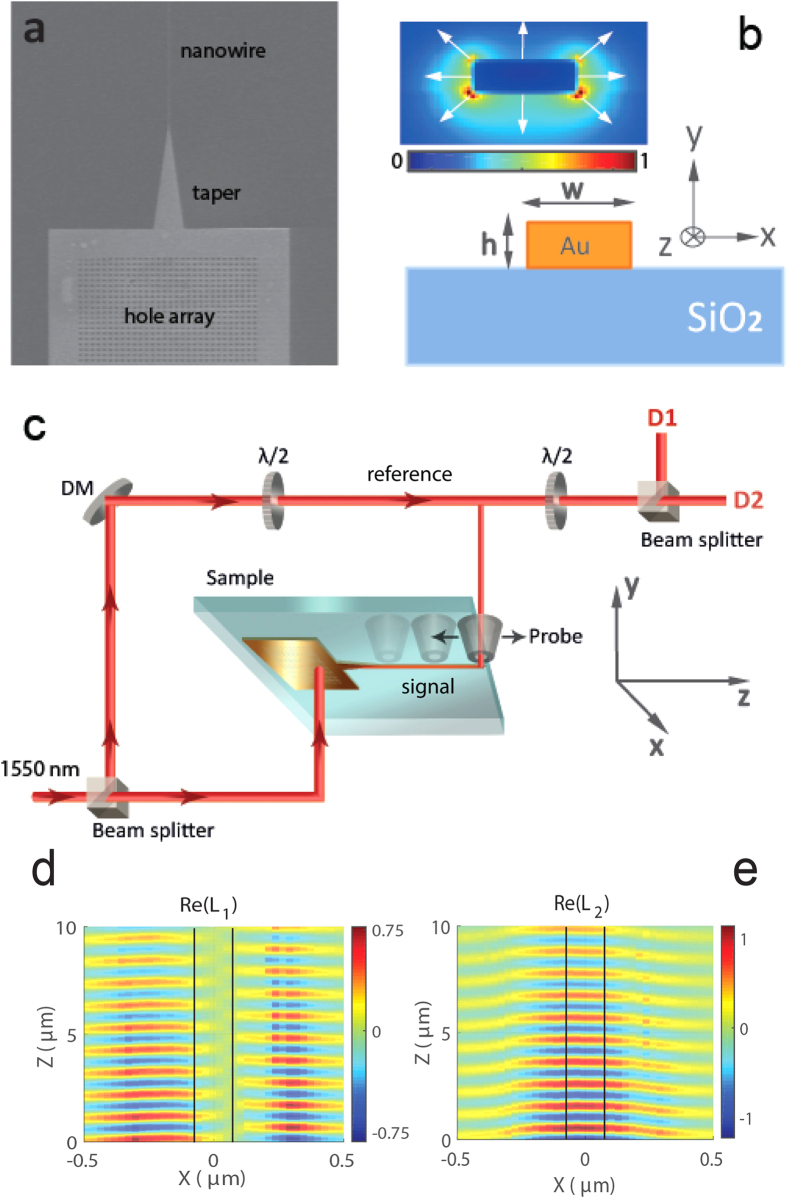
(**a**) Scanning electron microscope image of the Au strip nanowire sample, (**b**) Schematic of a gold nanowire placed on a glass substrate. The nanowire is *w* = 130 nm in width and *h* = 50 nm in height. The inset shows the normalized intensity of a plasmonic mode, with arrows indicating the direction of the transverse electric field. The calculation performed using the finite-difference time-domain method as discussed in Methods. (**c**) Schematic of the scanning near-field microscope setup: DM -dielectric mirror, D1 and D2 are detectors that record *x*- and *z*-polarized electric fields.(**d–e**) Real parts of complex signals *L*_1_ and *L*_2_ measured by detectors D1 and D2, respectively, for the probe scanning in the *xz*-plane at the height 20 nm above the wire. The signal amplitudes are normalized to the maximum amplitude of *L*_2_. Black lines indicate edges of the nanowire.

The sample is presented in Fig. 1(a). The plasmonic nanostructure is fabricated in a 50 nm thick layer of gold, that is deposited on top of a glass substrate. The details of the sample fabrication are described in Methods. In brief, it consists of a 2D hole array, terminated by a 20 *μ*m long taper, which funnels into a 130 nm wide and 50 *μ*m long nanowire (schematic of the nanowire is presented in Fig. 1(b)). We use the hole array to excite surface plasmon polariton waves and direct them towards the taper. The distance between the holes and the hole radius is optimized for coupling of the incident CW light source at 1550 nm. The adiabatic taper converts the initial waveguide mode, which is mainly confined to the interface between the glass substrate and the bottom surface of the gold taper, into a Sommerfeld-like nanowire mode^28^. At the chosen width and height of the nanowire, there is only one mode supported by it. The calculated spatial distribution of the normalized intensity of the electric field in such a plasmonic mode is depicted in the inset of Fig. 1(b). This mode has an approximately ‘radial’ polarization in the *xy*-plane and is confined to the corners of the nanowire. The structure of this mode therefore resembles a Sommerfeld wave in a cylindrical metal wire and has been reported previously^15^.

The NSOM probe, sensitive to the electro-magnetic near field, collects an optical signal for each probe position above the sample. This signal is then delivered by the optical fiber to detectors D1 and D2 (Fig. 1(c)). Our near-field microscope allows the measurement of both amplitude and phase of the evanescent field by means of an interferometric approach, in which a signal collected from the sample is combined with a reference signal. By introducing polarization optics into the detection path of the setup, we are able to distinguish between the orthogonal components of the in-plane near field. As has been discussed previously^21^,^26^, each detector provides a signal representation of a combination of the in-plane electric and magnetic field components. We ensure that the signal resulting from *x*-oriented electric field above the nanowire, 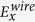, is collected by detector D1, and likewise 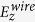 is detected by D2. Any signal that arises from *x*-oriented magnetic field, 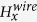, is detected by D2, and 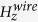 is picked up by D1.

Figure 1(d,e) present the real parts of complex signals *L*_1_ and *L*_2_, measured by the two detectors D1 and D2 respectively. As follows from the schematic of the experimental setup in Fig. 1(c), *L*_1_(**r**) and *L*_2_(**r**) result from the interference between the near-field signal at each position **r** and the reference signal, and can be written as 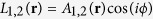, where *ϕ* is the phase difference between two arms of the interferometer. The height of the aperture probe for this set of measurements was kept approximately at 20 nm above the sample. We find that the *x*-polarized signal (*L*_1_) is anti-symmetric and has a node at *x* = 0, whereas the *z*-polarized signal (*L*_2_) is symmetric and has a non-zero magnitude at the center of the wire. This agrees with the description of the mode structure for nanoscale plasmonic nanowires reported previously^15^. The amplitude of the signal *L*_2_ is slightly larger than that of *L*_1_. The presence of a significant signal *L*_2_, which is associated with longitudinally-polarized fields, demonstrates the profound deviation of the highly confined nanowire mode from a transverse wave. Next we notice a tilt to the left side in the measurement data, which can be clearly seen in Fig. 1(d). This is due to the slight misalignment of the wire with regard to the scanning *z* axis, but it is not critical for the rest of the discussion.

**Figure 2:**
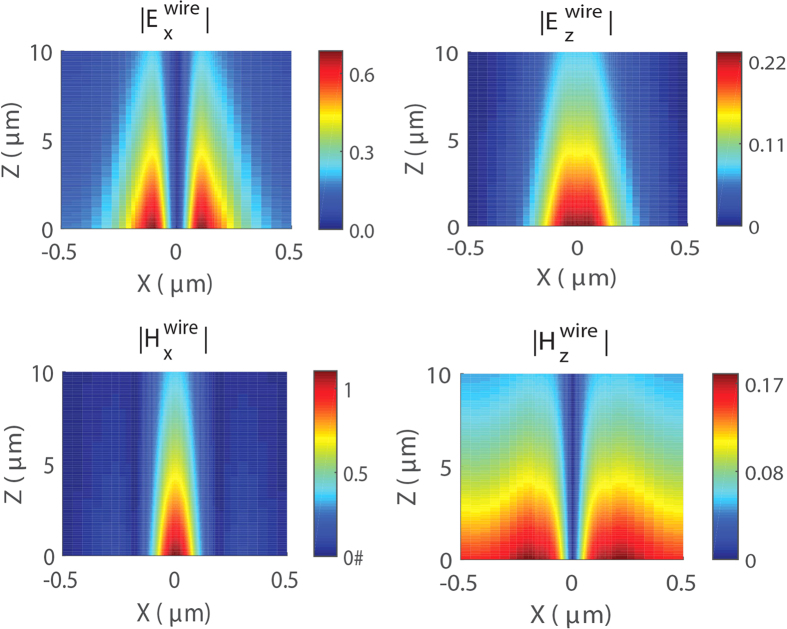
Simulated electric and magnetic field components in the *xz*-plane for a gold nanowire with a width of *w* = 130 nm. The field amplitudes are normalized to the maximum amplitude of  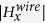 component.

Next we compare measured signals *L*_1_ and *L*_2_ to the fields obtained by numerical modeling of an SPP wave, propagating in a gold nanowire with the width *w* = 130 nm. For the simulation we use the material parameters of gold and glass at the wavelength of interest from a standard source^29^. We simulate the propagation of SPPs using the finite-difference time-domain (FDTD) method. More details on the simulations are presented in Methods. Figure 2 shows maps of calculated electric and magnetic field components in the *xz*-plane, 20 nm above the surface of the wire. From the amplitude and the phase (the latter not presented in Fig. 2) information we conclude that fields 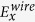 and 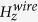 are antisymmetric, similar to the signal *L*_1_ (Fig. 1(d)). Fields 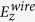 and 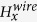, however, are symmetric with respect to the center of the wire *x* = 0, similar to the signal *L*_2_ (Fig. 1(e)). Magnetic fields in Fig. 2 are multiplied by the vacuum impedance 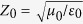, where *μ*_0_ and *ε*_0_ are vacuum permeability and permittivity, respectively. This allows us to bring fields to the same units of volt per meter for comparison. In calculations we find that the largest field component is the *x*-polarized magnetic field (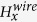), indicating that magnetic fields are not negligible. We also note that the distributions of the electric and magnetic fields share the same feature, such as symmetry, but are not exactly the same. For example, 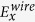 is more confined to the nanowire edges than 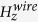. Additionally, 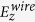 has two maxima, whereas 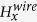 has only one maximum at the center of the wire. These observations together with the fact that a-NSOM has been previously shown to measure magnetic fields^21^,^27^, suggests that all in-plane electro-magnetic field components contribute to the image formation.

The question we have to answer next is how these fields combine to produce the signals *L*_1_ and *L*_2_. This is not a trivial question because we need to take into account the width of the nanowire (130 nm), the complex structure of the nanowire’s mode, and the diameter of the probe’s aperture (200 nm). Naively, we might expect the probe to act as an integrating filter with a Gaussian-like response. Due to the aperture being larger than the nanowire itself, such a filter applied to the intrinsic fields of the nanowire would lead to a blurring effect, such that the measured signals would have a larger spread in space than the intrinsic nanowire fields. Fortunately, there is no need for speculation as we can use a rigorous approach for the signal reconstruction based on the optical reciprocity theorem^30^. This is the subject of the next section.

### Optical reciprocity theorem and probe’s sensitivity to electric and magnetic fields near plasmonic nanowire

It is known that the optical reciprocity theorem can be used to explain near-field data collected during a measurement^30^,^31^. This theorem postulates that the detected signal from a source will be the same if the source and the detector are exchanged^32^. Recently, the reciprocity theorem has been successfully applied for the reconstruction of electro-magnetic fields above a *W*1-type PhCW^21^. In this article we will follow the same approach to explain the measurement of evanescent fields associated with the plasmonic nanowire mode.

**Figure 3:**
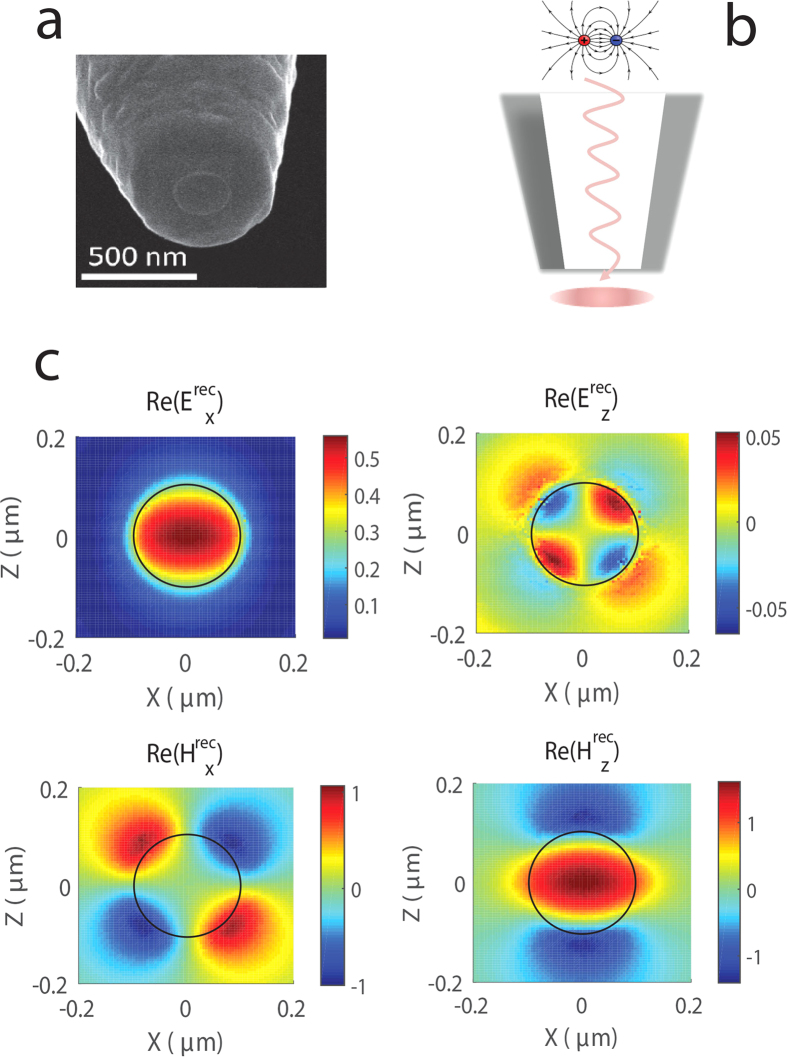
(**a**) SEM image of the aperture probe, (**b**) Illustration to the calculation of reciprocal fields: a dipole source placed at the detector position emits light that is imaged below the aperture, (**c**) Reciprocal fields in the *xz*-plane calculated below the aperture probe, arising from the *x*-oriented dipole source. The field amplitudes are normalized to the maximum amplitude of 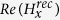 component. Black circle indicates a cross-section of the aperture.

The reciprocity theorem relates the fields measured by the probe to the fields that would be transmitted through the probe if a dipole source were placed at the location of the detector. Mathematically, when we neglect any back-action from the probe, the expected signals can be expressed as


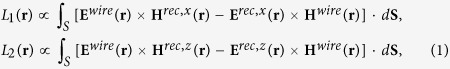


where we take **S** to be in the *xz*-plane. Here **r** is the position of the probe tip above the nanowire. 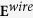 and 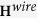 indicate the calculated fields above the gold nanowire due to the propagating plasmonic mode. 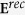 and 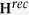 are, the so-called, reciprocal fields, which are calculated just below the probe and which result from an *x*- or *z*-polarized dipole source placed at the detector position^32^. We note that 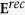 and 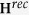 are virtual fields as they are not present in the actual measurement. They, however, are very useful for calculations and can be taken to reflect the components of the probe’s complex response function. The concept of the reciprocal fields is illustrated in Fig. 3, where (a) shows SEM image of the actual microscope tip and (b) illustrates the model which we use to calculate reciprocal fields. Eq. (1) suggests that the reciprocal magnetic fields reflect the sensitivity of the probe to the electric field components of the wire


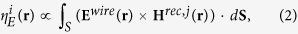


as well as the reciprocal electric fields reflect the probe’s sensitivity to the wire’s magnetic fields


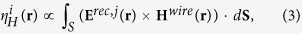


where *i* = 1, 2 denotes the detector number and *j* = *x, z* shows the respective orientation of the dipole source. For *i* = 1 the dipole is oriented along *x* (*j* = *x*), whereas it is oriented along *z* (*j* = *z*) for *i* = 2.

To calculate the reciprocal fields 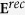 and 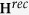 we use the finite element method and approximate the near-field probe by a glass hole with a diameter of *d* = 200 nm in a 150 nm aluminum layer. This approximation is justified, since the thickness of the aluminum layer on the probe is large enough to have no significant electro-magnetic fields outside the metal coating when the source and the detector are exchanged. It has also been shown that the fields produced by such a hole closely resemble the transmitted fields of a real aperture probe^33^. As strongly suggested by recent works^34^,^35^, the sensitivity of an aperture probe can be controlled through the thickness of the metal coating. This situation is particularly important for probes with thin metal claddings. In this work, however, the metal cladding is relatively thick and we focus on the extent to which the detected field components can be separated so that a local description of the field coupling to the probe is valid. From Eq. (1) and the definition of *d***S** = **y***dS* we conclude that the only non-zero terms under the integral are those constructed by the reciprocal and wire fields in the *xz*-plane, since the vector product of the out-of-plane fields with *d***S** is zero.

Figure 3(c) shows the calculated reciprocal fields 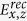 and 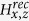 below the tip of the probe. The magnitudes of these fields are normalized to the maximum of 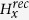 to simplify the comparison. We see that the distribution of 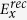 resembles a slightly squeezed Gaussian beam, whereas the other three components have a more complex structure. It would therefore be wrong to expect the probe to behave as a simple integrating filter. We also note that the reciprocal magnetic components 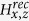 are stronger than the electric counterparts. Recall that the magnetic near-field component of the wire field 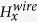 is also the strongest among the rest of wire fields. This is an important observation and we expect it to play a role in the probe’s sensitivity to the electric and magnetic fields.

After finding the reciprocal fields we can now convolve them with the wire fields using the recipe given by Eq. (1). By doing so we are able to calculate the signals *L*_1_ and *L*_2_ at every position of the probe in the *xz*-plane above the nanowire.

**Figure 4:**
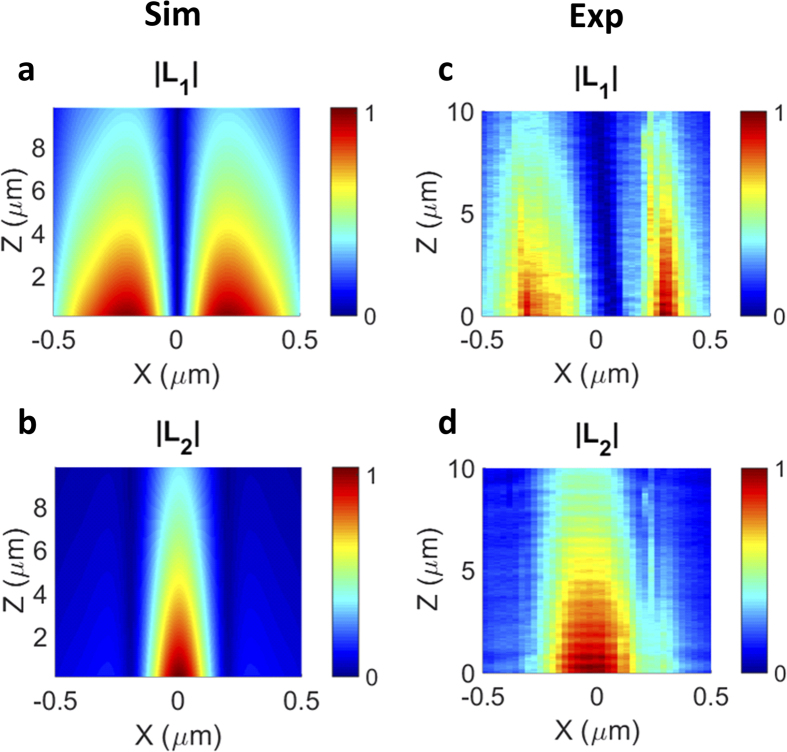
Reconstructed (**a,b**) and measured (**c,d**) signals at detectors D1 and D2. The amplitudes of all signals are normalized to their maxima.

The result of this numerical procedure is presented in Fig. 4, where the calculated signals using the reciprocity theorem are shown in (a,b), and the measured signals are given in (c,d) for the comparison. We notice that the calculated signals (Fig. 4(a,b)) exhibit a very good agreement with the measurement (Fig. 4(c,d)). The slight asymmetry in the measured data can be attributed to a small degree of polarization mixing, i.e. non-perfect separation of orthogonal polarizations in the experiment. This is, however, hard to avoid since the polarization of light in an optical fiber is susceptible to drifts due to temperature and stresses^36^.

One of the important conclusions can be drawn after analyzing Fig. 4(a,c): the field *L*_1_ (as well as its calculated version) looks markedly different from the individual field components 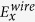 and 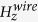. Apart from the width of the lobes, the most striking difference is the distance between the lobes. In fact, the separation between the lobes (measured as the distance between the field maxima) in Fig. 4(c) is close to 600 nm, which is almost 3 times the separation between the lobes of the field 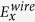 in Fig. 2. In fact, it can be shown that the signal *L*_1_ in these measurements cannot be constructed as a linear superposition of the respective electric and magnetic field components, 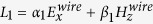, where 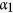 and 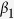 are complex constants. This finding is contrary to the experimental observations while mapping the electric and magnetic fields above a photonic crystal waveguide^21^. Apparently, the structure of the fields around a nanophotonic structure combined with the complexity of the near-field coupling to the probe, see e.g. Eq. (1), makes that the simple separation in a superposition of individual **E** and **H** components can only be performed for certain conditions.

Another piece of information that we are able to retrieve using the reciprocity theorem is the sensitivity of the probe to the electric and magnetic field components of the nanowire’s near field. From Eq. (2) and (3) it follows that the probe sensitivity is not only represented by the structure and magnitude of the reciprocal fields, but also depends on the fields the probe is measuring. This means that, in principle, the probe’s sensitivity can differ depending on which structure it measures: a plasmonic nanowire, a subwavelength hole or a photonic crystal. This is an important property of the near-field imaging that originates from the complexity of the collection process as is evident from Eq. (1). Using Eq. (2) and (3) we finally calculate the sensitivity ratio to the magnetic and electric fields, averaged across the calculation area, to be 
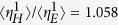
 for the detector D1 and 
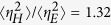
 for the detector D2. We find that the ratio of sensitivities varies for each detector, but overall is slightly larger than 1, meaning that the probe is slightly more sensitive to the magnetic near field than to the electric one. This could be explained by the stronger expected magnetic response of the probe, that is evident if we compare magnitudes of the field components in Fig. 3.

## Discussion

Our analysis of the near field, associated with the propagating mode in a plasmonic nanowire, has shown that the contribution of the magnetic field to the measured signals cannot be neglected, because it is equal to or in some instances even larger than the contribution of the electric field^20^,^27^. We have also determined that the probe sensitivity to the magnetic field is approximately equal to the probe sensitivity to the electric field. A similar situation was reported for the near-field imaging of PhCWs, in which the probe sensitivity ratio 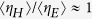 was found for most of the probe sizes and independent of the height of the probe above the crystal^21^.

It might seem, however, a puzzling outcome that the ratio of sensitivities in our system differs for the two detectors (
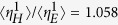
 and 
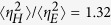
), albeit modestly. At first glance, it seems nonphysical as the probe used in the measurements was cylindrically symmetric. Moreover, in the simulations the probe was assumed to be symmetric, which was reflected in the calculation of the reciprocal fields (the reciprocal fields calculated for z-polarized dipole source are a projection-swap version of the fields calculated for x-polarized dipole source). Thus, the different value of the sensitivity ratio for the two detectors would seem impossible, if the sensitivity of the probe were to depend on the probe’s properties only. The sensitivity of the probe, however, is also determined by the properties of the fields it is measuring. This circumstance allows for a perfectly symmetric probe in a perfectly symmetric detection scheme to have different sensitivity ratios in the two orthogonal field directions.

As we discussed earlier, due to the complex nature of the image formation process, the link between the local intrinsic field of the nanowire and the signal measured at this same location is nontrivial. Therefore, it is not possible to represent the signals *L*_1_ and *L*_2_ as a linear superposition of the nanowire electric and magnetic fields, and consequently separate **E** and **H** fields in the measurement. This is in contrast to the near-field measurement of PhCWs^21^,^26^ and of a plasmonic aperture^20^ reported previously. In the situation with photonic crystals it was possible to fit the measurement at nine different heights above the crystal by forming linear superpositions of the electric and magnetic fields using only 2 fitting parameters, the probe sensitivities. In those experiments, the period of the photonic crystal was 420 nm and the hole diameter was 240 nm^21^, somewhat larger than the nanowire dimensions and comparable to the probe’s aperture. Another point of difference in the present work is the structure of the wire’s mode with two of the field projections (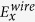 and 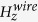) being antisymmetric. We expect that the field separation problem can be solved if a smaller probe is used. In principle, probes with aperture diameters of approximately a 100 nm can be easily fabricated. Such probes, however, prove to be impractical since they typically have a very poor transmission and coupling efficiency. Another solution could be to use a scattering probe^37^, with a diameter on the order of tens of nanometers^38^, instead of a collecting aperture probe. Care must be taken, however, to ensure that the interaction of the scattering probe with the sample is minimal and can be described by the first order perturbation theory^39^.

In summary, we have performed near-field imaging of plasmonic nanowires using a home-built polarization- and phase-sensitive near-field microscope operating in a collection mode. The analysis of the measured signals has shown that the collected light is the combination of electric and magnetic fields, and the magnetic field contribution is significant. We have developed a model of the image formation in our microscope, based on the optical reciprocity theorem. This theorem allows us to include the response of the aperture probe into consideration and calculate the signals measured by the detectors. We find a very good agreement between our measurement and the modeling results. Overall, our findings indicate the importance of a careful analysis of the near-field measurement data and consideration of both electric and magnetic contributions of the near field. The identification of the strong magnetic contribution to the near-field signals measured in vicinity of plasmonic nanostructures paves the way towards studies of magnetic effects at optical frequencies^40^,^41^,^42^.

## Methods

Nanowire, waveguide taper and hole array are fabricated in a 50 nm thick Au film on the BK7 glass substrate by electron beam lithography in a bilayer PMMA resist followed by liftoff. The Au is evaporated through resistive heating, resulting in improved uniformity and smoothness of the layer. The hole array has a pitch of 1 *μ*m and a hole diameter of roughly 0.5 *μ*m. The nanowire length is approximately 50 *μ*m.

For the numerical modeling we used FDTD Solutions by Lumerical, to simulate the propagation of SPPs in our nanowire structure. We chose perfectly matched layers as the condition on all boundaries of the model. The nanowire mode was calculated using the Mode Expansion Monitor. This mode then was propagated along the length of the nanowire using a home-build code in Matlab to obtain in-plane field maps shown in Fig. 2. The simulation took into account the change of the tip’s height during the measurement scan. Since the scan was made in the ‘constant distance’ mode, the probe followed the topography while scanning above the sample. For the simulation of the reciprocal fields we used the finite element method implemented in COMSOL, similar to our previous work^21^. The overlap integrals associated with Eq. (1, 2, 3) were calculated using a home-build code in Matlab.

## Additional Information

**How to cite this article**: Kabakova, I. V. *et al.* Imaging of electric and magnetic fields near plasmonic nanowires. *Sci. Rep.* **6**, 22665; doi: 10.1038/srep22665 (2016).
